# Hepatitis C Virus (HCV) Clearance Cascade for Persons With Human Immunodeficiency Virus (HIV)/HCV Coinfection Using Health Department Surveillance Data Among 7 US Jurisdictions Highlights the Role of HIV Care Engagement

**DOI:** 10.1093/ofid/ofaf412

**Published:** 2025-08-07

**Authors:** Maximilian Wegener, Ralph Brooks, Lisa Nichols, Frederick L Altice, Merceditas Villanueva

**Affiliations:** Yale School of Medicine, Department of Internal Medicine, Section of Infectious Disease, HIV/AIDS Program, New Haven, Connecticut, USA; Yale School of Medicine, Department of Internal Medicine, Section of Infectious Disease, HIV/AIDS Program, New Haven, Connecticut, USA; Yale School of Medicine, Department of Internal Medicine, Section of Infectious Disease, HIV/AIDS Program, New Haven, Connecticut, USA; Yale School of Medicine, Department of Internal Medicine, Section of Infectious Disease, HIV/AIDS Program, New Haven, Connecticut, USA; Yale School of Medicine, Department of Internal Medicine, Section of Infectious Disease, HIV/AIDS Program, New Haven, Connecticut, USA

**Keywords:** coinfection, HCV, HIV, public health surveillance, viral clearance cascade

## Abstract

**Background:**

Persons with human immunodeficiency virus (HIV) coinfected with hepatitis C virus (HCV) experience worse health outcomes compared to HCV-monoinfected individuals and can benefit from highly effective direct-acting antivirals (DAAs). Despite their availability, DAAs have not been comprehensively implemented to achieve the 80% national viral cure target. Understanding the HCV care continuum for people with HIV is critical for addressing public health intervention gaps. We worked with 7 diverse health department jurisdictions to implement a standardized Centers for Disease Control and Prevention HCV clearance cascade for coinfected people with HIV.

**Methods:**

We developed data collection tools upon matching HIV and HCV surveillance datasets, automating HCV clearance cascade generation for a cohort of coinfected persons from 31 December 2019 through 31 December 2021. We conducted multivariable analysis to assess progress toward elimination targets and identify risk factors for poor HCV clearance.

**Results:**

Combined clearance cascades showed 31.6% viral cure/clearance at baseline and 42.4% at the study's end. Black/African American persons exhibited significantly lower odds of cure/clearance compared to White individuals (adjusted odds ratio [aOR], 0.83; *P* = .03). Increased viral cure/clearance rates were seen in men who have sex with men compared to heterosexuals (aOR, 1.46; *P* = .004). Those who had HIV viral suppression were more likely to have cleared HCV (aOR, 2.19; *P* < .0001).

**Conclusions:**

HCV viral cure/clearance rates in this coinfected population, while better compared to published rates for persons with HCV monoinfection, remain far below strategic national target goals. Optimal HIV care engagement was associated with improved HCV outcomes, suggesting that public health strategies that build on established clinical models and public health infrastructure for HIV can be leveraged to improve HCV outcomes.

## Teaser

Using surveillance data to create jurisdictional HCV clearance cascades, we found that HCV clearance rates for HIV/HCV coinfected persons remain below national HCV elimination targets. HIV viral suppression correlated with HCV clearance, suggesting the importance of leveraging HIV-focused clinical/public health resources.

## Graphical Abstract

**Graphical Abstract:**
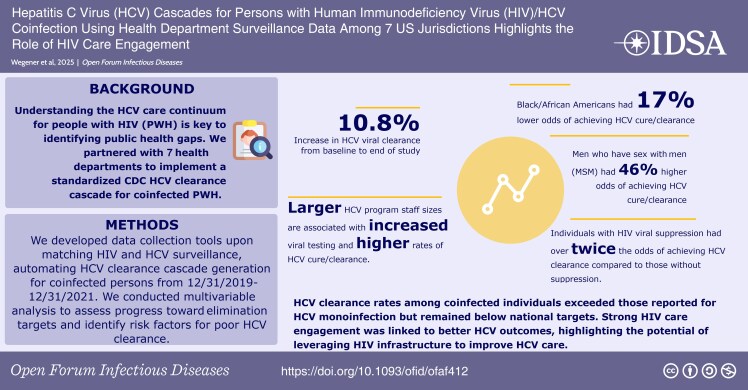
This graphical abstract is also available at: https://tidbitapp.io/tidbits/hcv-clearance-cascades-for-persons-with-hiv-hcv-coinfection-using-health-department-surveillance-data-among-seven-u-s-jurisdictionshighlights-the-role-of-hiv-careengagement?utm_campaign=tidbitlinkshare&utm_source=ITP

Chronic hepatitis C virus (HCV) infection is a major cause of liver-related morbidity and death. Persons with human immunodeficiency virus (HIV) and HCV coinfection experience worse clinical outcomes than those with HCV monoinfection even in the era of highly effective antiretroviral treatment [1–5]. Direct-acting antivirals (DAAs) have revolutionized HCV treatment, curing and preventing long-term consequences of infection with treatment efficacy of >95% for HCV-monoinfected and HCV/HIV-coinfected persons [6, 7]. National and global plans to eliminate HCV through enhanced testing and treatment, including for persons with HIV/HCV coinfection, have been developed [8, 9].

Public health interventions to promote HCV elimination in this population require an understanding of the HCV continuum of care. A key strategy is the development of health department–level, surveillance-based HCV clearance cascades, a tool to help jurisdictions visualize diagnosis and treatment milestones, identify gaps, and monitor progress toward national elimination goals. The Centers for Disease Control and Prevention's (CDC) *Guidance for Jurisdictional Hepatitis C Elimination Strategic Planning* serves as a roadmap for health department jurisdictions [9]. The CDC developed public health guidance for the HCV clearance cascade, allowing standardized use of HCV surveillance data as well as other HCV databases [10].

Feasibility of this guidance was shown using a national commercial laboratory database [11]. Of 1.7 million persons with HCV, 34% achieved clearance, with lower rates among persons aged 20–39 years and those with Medicaid or self-pay insurance. Subsequent analyses also show state-level variations in HCV clearance ranging from 10% to 51% [12]. However, HCV clearance cascades using health department–based surveillance data have not been widely implemented. This highlights gaps in HCV surveillance infrastructure due partly to historically low federal investment. In contrast, HIV surveillance infrastructure is significantly more robust with the CDC-developed Enhanced HIV/AIDS Reporting System (eHARS) that assists health departments with HIV reporting, data management, and analysis.

We capitalized on the availability of well-established HIV surveillance data to pilot a matching approach to HCV surveillance data to create an HCV clearance cascade for coinfected persons. We previously used this approach in Connecticut showing that among 1361 coinfected persons there was a 38.8% clearance rate (as of 31 December 2019) [13]. Notably, those with undetectable HIV viral loads were more likely to achieve HCV cure, with no significant demographic differences in clearance rates [13].

The HIV and HCV epidemics vary in different parts of the United States (US) [14], leading to variations in the HCV care continuum. In this project, we aimed to examine the scope of HCV care in coinfected populations among diverse health department jurisdictions in the US. This allows for examination of unique challenges and strengths that exist among different health department jurisdictions for achieving HCV outcomes among persons with HIV/HCV coinfection.

## METHODS

### Participants

A Health Resources and Services Administration (HRSA) grant (“Leveraging a Data to Care Approach to Cure Hepatitis C within the Ryan White HIV/AIDS Program: A Multisite Partnership”) funded the Yale School of Medicine as a technical assistance provider to 7 different health department jurisdictions (Arizona Department of Health Services, Connecticut Department of Public Health, Florida Department of Health in Orange County [FLOC], Kentucky Department of Public Health, Michigan Department of Health and Human Services, Southern Nevada Health District, and Puerto Rico Department of Health). These jurisdictions were selected for their diverse geographic and population characteristics. Participating jurisdictions had to meet specific criteria, including a reliable HIV surveillance database; a longitudinal HCV laboratory result database; and processes for collecting HCV polymerase chain reaction (PCR)–positive and –negative laboratory data results.

### Jurisdictional Baseline Assessment Surveys

Surveys were administered through REDCap (a secure web-based electronic data capture tool) and consisted of 215 questions on personnel, data systems, HIV and HCV surveillance programs, and data matching (Supplementary Tables 2 and 3). Data from these surveys were also used to assess each jurisdiction's capacity to create HCV clearance cascades for their HIV/HCV-coinfected populations, from which a baseline assessment outlining minimum requirements to create HCV clearance cascades (Supplementary Table 1) was completed. The surveys also collected data for identifying differential capacities in Data to Care (D2C) activities, opioid use disorder programs, and Ryan White–funded programs.

### Jurisdiction Surveillance Programs and Data Sources

Each jurisdiction used eHARS for HIV surveillance but distinct databases for HCV. Supplementary Table 2 lists details for each jurisdiction's HCV surveillance databases and the software used to match HIV and HCV databases. The table also presents full-time equivalent (FTE) staffing per surveillance program, with a median of 4 FTEs for HIV and 1.25 for HCV. In 1 jurisdiction (Puerto Rico), the lack of an HCV surveillance database necessitated the use of CAREWare (a free data system developed by HRSA for Ryan White–funded service delivery) as an alternative [15].

### Data Collection

Project data were aggregated by jurisdiction, including demographic and clinical details on HIV, HCV, and coinfected populations using the project's standardized Jurisdictional Data Collection Tool [16]. The baseline cohort included persons with HIV/HCV coinfection based on prevalent eHARS as of 31 December 2019 matched to the HCV database. Quarterly updated data submissions tracked cascade progress over 2 years (31 December 2019–31 December 2021) at 6-month intervals. With each quarterly update, jurisdictions updated the starting denominator by removing deceased or relocated persons. HCV care status was assessed based on specific HCV test result dispositions for each clearance cascade step [10, 11, 13], and jurisdictions submitted data via REDCap. Training videos were developed to address baseline assessment gaps covering topics related to data cleaning, updating HCV surveillance data, database matching (Match*Pro) [17], cascade dispositions, cascade inclusion and exclusion, and creating and using the HCV clearance cascade. These videos are available as mini-modules with an accompanying *Written Companion and Implementation Manual* on the HRSA Target HIV website [16].

### HCV Clearance Cascade Creation

The HCV clearance cascade includes 5 steps: ever infected; viral testing (PCR testing); initial infection (ie, chronic HCV or PCR positive); cured or cleared; and persistent infection or reinfection [10]. Jurisdictions submitted their laboratory disposition data using the Jurisdictional Data Collection Tool, auto-generating their HCV clearance cascade. Operational methodologies, including personnel responsible for generating cascades, varied by jurisdiction (Supplementary Table 3).

### Cascade Outcomes

There were 2 main outcomes: viral testing (Step 2) and cure/clearance (Step 4). Variables (demographic and clinical status) associated with each outcome were analyzed.

### Statistical Analysis

Data analysis was done in collaboration with the Yale Center for Analytic Sciences. We performed descriptive analysis to examine the distribution of demographic data and clinical information for aggregated jurisdiction baseline data. Pearson χ^2^ tests assessed associations between jurisdictional HIV and HCV data. Conditional proportions for each demographic variable were calculated within each cascade step.

Using SAS version 9.4 software, we conducted univariable logistic regression to assess associations between demographic variables and the 2 primary outcomes and to calculate odds ratios (ORs) and 95% confidence intervals (CIs). To account for jurisdictional variability, we also performed a multivariable logistic regression model, which included the demographic variables while adjusting for jurisdiction as a covariate. This adjustment isolated the demographic variables’ effects while accounting for geographic differences. Significance was determined using a 2-sided *P* value with a threshold of .05.

Additionally, we conducted a separate comparative analysis using R software (R Core Team, 2021) to examine jurisdictional-level differences in viral testing and cure/clearance. These were descriptive analyses using 2 × 2 contingency tables, and ORs with 95% CIs were calculated using the Wald method. These were not regression-based models but provided comparative measures between jurisdictions.

### Patient Consent Statement

The Yale Human Investigations Committee reviewed the research and deemed it exempt from signed consent requirements because it used de-identified data (Institutional Review Board number 2000025899).

### Participating Jurisdiction Approval

Each of the 7 participating jurisdictions reviewed and approved the manuscript for accuracy and compliance with their internal policies regarding publication.

## RESULTS

### Demographics

Table 1 presents aggregate baseline demographic and clinical data for persons with HIV, HCV, and HIV/HCV coinfection across the 7 health department jurisdictions (detailed demographics by jurisdiction are shown in Supplementary Table 4*A–C*). Overall, the number of persons with HCV is 6.6 times greater than those with HIV; 10.2% of persons with HIV and 1.5% with HCV are coinfected. Racial distribution did not differ significantly. Hispanic/Latino persons were more prevalent in the HIV (29.9%) and coinfected groups (36.7%) compared to the HCV group (11.9%). More males were in the HIV (78.5%) and coinfected groups (75.2%) compared to the HCV group (61.5%). Major HIV transmission risk factors differed, with men who have sex with men (MSM) status being the major risk for HIV (59.0%) and injection drug use (IDU) for coinfection (44.0%). Coinfected individuals were more likely to have had an HCV test within the past year (34.6%) compared to those with HCV alone (20.2%). No differences were seen in HIV viral suppression status between the HIV and coinfection groups.

**Table 1.** Overall Demographics for Persons With Human Immunodeficiency Virus (HIV), Hepatitis C Virus (HCV), or HIV/HCV Coinfection Among 7 Different Health Department Jurisdictions

Demographic Variable	HIV Monoinfected (N = 77 123)^a^		HCV Monoinfected (N = 512 125)^a^		HIV/HCV Coinfected (N = 7832)^a^		*P* Value
	No.	%	No.	%	No.	%	
Race							
White	47 755	61.9%	369 649	72.2%	5269	67.3%	.73
Black or African American	25 592	33.2%	106 068	20.7%	2232	28.5%	
Asian/Native Hawaiian/Pacific Islander	1245	1.6%	4904	1.0%	66	0.9%	
American Indian/Alaska Native	882	1.2%	10 693	2.1%	97	1.2%	
Other	1648	2.1%	20 810	4.0%	167	2.1%	
Ethnicity							
Hispanic/Latino	23 047	29.9%	60 816	11.9%	2872	36.7%	.**0002**
Non-Hispanic/Latino	54 076	70.1%	451 309	88.1%	4960	63.3%	
Age group, y							
<18	365	0.5%	3485	0.7%	5	0.1%	.42
18–25	3063	4.0%	23 825	4.7%	76	1.0%	
26–35	13 589	17.6%	77 766	15.2%	633	8.1%	
36–45	14 628	19.0%	99 073	19.3%	1137	14.5%	
46–55	20 978	27.2%	124 801	24.4%	2159	27.5%	
56–65	17 790	23.0%	116 950	22.8%	2888	36.9%	
66–75	5604	7.3%	51 186	10.0%	871	11.1%	
>75	1106	1.4%	15 038	2.9%	63	0.8%	
Sex at birth							
Male	60 554	78.5%	314 785	61.5%	5886	75.2%	.**02**
Female	16 569	21.5%	197 340	38.5%	1946	24.8%	
HIV transmission type							
Male-male sexual contact	45 490	59.0%	…	…	1969	25.1%	**<**.**0001**
Injection drug use	5750	7.4%	…	…	3442	44.0%	
Male-male sexual contact and injection drug use	3374	4.4%	…	…	859	11.0%	
Heterosexual contact (male-female)	20 283	26.3%	…	…	1405	17.9%	
Other	2226	2.9%	…	…	158	2.0%	
HIV suppression status (within 12 mo)							
Viral load undetectable (<200 copies/mL)	66 674	86.4%	…	…	6782	86.6%	1.0
Viral load detectable (≥200 copies)	10 449	13.6%	…	…	1050	13.4%	
Time since last HIV viral load, CD4, or HIV genotype							
≤6 mo	46 673	60.5%	…	…	5891	75.2%	.17
>6 to 12 mo	17 091	22.2%	…	…	1044	13.3%	
>12 to 18 mo	2941	3.8%	…	…	234	3.0%	
>18 mo	10 419	13.5%	…	…	663	8.5%	
Time since first available HCV^+^ result (Ab or PCR)							
<1 y	…	…	62 084	12.1%	574	7.3%	.59
1 to <2 y	…	…	45 841	9.0%	590	7.5%	
2 to <5 y	…	…	116 227	22.7%	1626	20.8%	
≥5 y	…	…	287 973	56.2%	5041	64.4%	
Time since most recent HCV (Ab or PCR) test							
<1 y	…	…	103 406	20.2%	2708	34.6%	.**002**
1 to <2 y	…	…	53 233	10.4%	1246	15.9%	
2 to <5 y	…	…	101 228	19.8%	2037	26.0%	
≥5 y	…	…	254 253	49.6%	1841	23.5%	

Abbreviations: Ab, antibody; HCV, hepatitis C virus; HIV, human immunodeficiency virus; PCR, polymerase chain reaction.

^a^These 3 groups are mutually exclusive.

Bolded values indicate statistically significant *P*-values (*P* < .05).

### Longitudinal HCV Clearance Cascades for the HIV/HCV Coinfection Cohort (Baseline and Quarter 4)

**Figure 1.**
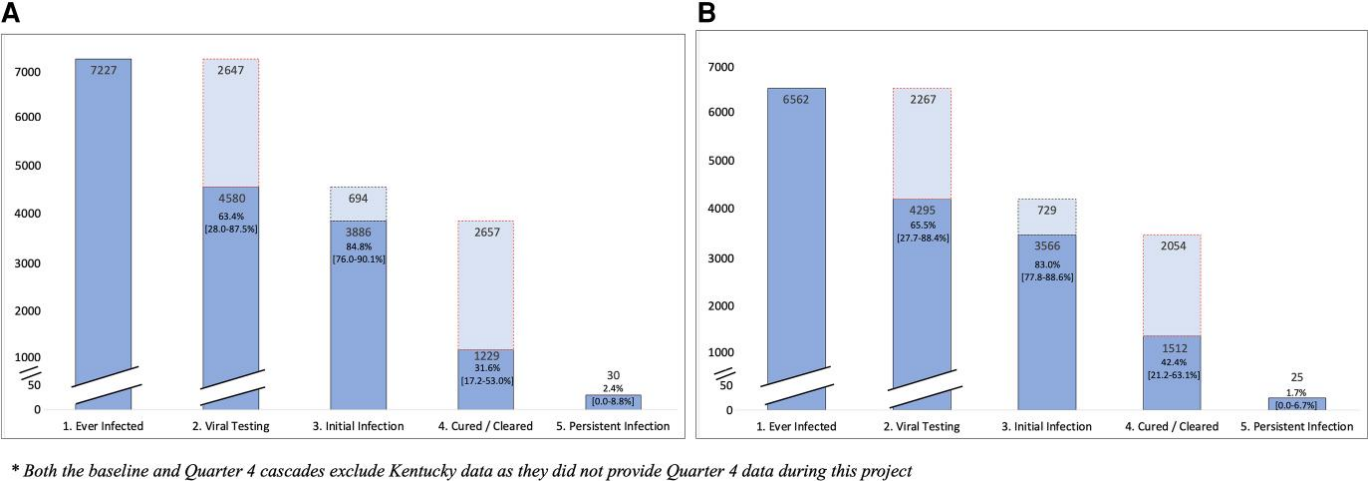
Combined hepatitis C virus clearance cascade comparison (6 jurisdictions) for baseline (*A*) (as of 31 December 2019) and Quarter 4 (*B*) (as of 31 December 2021). Both the baseline and Quarter 4 cascades exclude Kentucky data as they did not provide Quarter 4 data during this project.

All jurisdictions created baseline HIV/HCV-coinfected HCV clearance cascades, but only 6 jurisdictions provided longitudinal cascades up to Quarter 4 (Q4) (Figure 1). The number of coinfected decreased from 7227 at baseline (Figure 1*A*) to 6562 at Q4 (Figure 1*B*), after excluding deceased and out-of-jurisdiction individuals. Viral testing (Step 2) increased from 63.4% to 65.5% and viral clearance (Step 4) from 31.6% to 42.4%. Q4 viral clearance rates ranged between 21.2% and 63.1% (Supplementary Table 5).

Table 2 shows, by demographic subgroup, the conditional proportions for each cascade step, where each step’s proportion uses the count (N) from the prior step as the denominator. Black/African American individuals had a slightly higher viral testing rate (72.8%) compared to all other races. Initial infection rates were similar across races. Cure/clearance rates were highest among those >45 years old (>42%) and among individuals with IDU (44.6%) and MSM (43.5%) as HIV risk factors. Undetectable HIV viral loads were associated with higher cure/clearance rates compared to detectable viral loads (44.5% vs 27.2%). Shorter time since last HIV test was associated with higher HCV viral testing and cure/clearance rates, while the longest time since most recent HCV test had the lowest cure/clearance rates. Persistent infection or reinfection was generally low (<6%) across all demographics.

**Table 2.** Conditional Proportions for the Laboratory-Based Hepatitis C Virus Clearance Cascade by Subpopulation (as of 31 December 2021)

Variable	1. Ever Infected	2. Viral Testing		3. Initial Infection		4. Cured/Cleared		5. Persistent Infection/Reinfection	
	No.	No.	% (2/1)	No.	% (3/2)	No.	% (4/3)	No.	% (5/4)
	6562	4295	65.5%	3566	83.0%	1512	42.4%	25	1.7%
Race									
White	3951	2433	61.6%	1999	82.2%	825	41.3%	18	2.2%
Black or African American	2234	1627	72.8%	1369	84.1%	627	45.8%	6	1.0%
Asian/Native Hawaiian/Pacific Islander	81	46	56.8%	37	80.4%	17	45.9%	0	0.0%
American Indian/Alaska Native	111	72	64.9%	64	88.9%	12	18.8%	0	0.0%
Other	185	118	63.8%	97	82.2%	31	32.0%	1	3.2%
Ethnicity									
Hispanic/Latino	2653	1427	53.8%	1163	81.5%	481	41.4%	9	1.9%
Non-Hispanic/Latino	3909	2868	73.4%	2403	83.8%	1031	42.9%	16	1.6%
Age group, y									
<26	61	33	54.1%	25	75.8%	5	20.0%	1	20.0%
26–35	453	299	66.0%	238	79.6%	78	32.8%	2	2.6%
36–45	922	531	57.6%	460	86.6%	185	40.2%	4	2.2%
46–55	1878	1215	64.7%	989	81.4%	418	42.3%	6	1.4%
56–65	2472	1703	68.9%	1419	83.3%	640	45.1%	11	1.7%
>65	776	514	66.2%	435	84.6%	186	42.8%	1	0.5%
Sex at birth									
Male	4919	3244	65.9%	2721	83.9%	1150	42.3%	23	2.0%
Female	1643	1051	64.0%	845	80.4%	362	42.8%	2	0.6%
HIV transmission type									
Male-male sexual contact	1635	1060	64.8%	858	80.9%	373	43.5%	7	1.9%
Injection drug use	2895	2061	71.2%	1755	85.2%	782	44.6%	5	0.6%
Male-male sexual contact and injection drug use	669	501	74.9%	418	83.4%	154	36.8%	9	5.8%
Heterosexual contact (male-female)	1265	614	48.5%	486	79.2%	185	38.1%	3	1.6%
Other	99	59	59.6%	48	81.4%	19	39.6%	1	5.3%
HIV suppression status (within 12 mo)									
Viral load undetectable (<200 copies/mL)	5721	3774	66.0%	3129	82.9%	1393	44.5%	22	1.6%
Viral load detectable (≥200 copies)	841	521	62.0%	437	83.9%	119	27.2%	3	2.5%
Time since last HIV viral load, CD4, or HIV genotype									
≤6 mo	3829	2850	74.4%	2348	82.4%	1172	49.9%	15	1.3%
>6 to 12 mo	803	595	74.1%	516	86.7%	184	35.7%	5	2.7%
>12 to 18 mo	255	177	69.4%	135	76.3%	44	32.6%	0	0.0%
>18 mo	1675	672	40.1%	567	84.4%	112	19.8%	5	4.5%
Time since first available HCV^+^ result (Ab or PCR)									
<1 y	21	16	76.2%	14	87.5%	5	35.7%	0	0.0%
1 to <2 y	22	16	72.7%	15	93.8%	10	66.7%	0	0.0%
2 to <5 y	977	595	60.9%	432	72.6%	211	48.8%	5	2.4%
≥5 y	5543	3668	66.2%	3105	84.7%	1286	41.4%	20	1.6%
Time since most recent HCV (Ab or PCR) test									
<1 y	1635	1305	79.8%	1050	80.5%	630	60.0%	10	1.6%
1 to <2 y	533	463	86.9%	368	79.5%	231	62.8%	1	0.4%
2 to <5 y	1938	1343	69.3%	1076	80.1%	468	43.5%	10	2.1%
≥5 y	2456	1183	48.2%	1072	90.6%	183	17.1%	4	2.2%

Abbreviations: Ab, antibody; HCV, hepatitis C virus; HIV, human immunodeficiency virus; PCR, polymerase chain reaction.

### Predictors of HCV Viral Load Testing for the HIV/HCV Coinfection Cohort (Cascade Step 2)

Table 3 presents the univariable and multivariable analyses. Females and American Indian/Alaska Native, Asian/Pacific Islander, and Hispanic/Latino individuals had lower odds of HCV viral testing while those aged >56 years and those having IDU and MSM/IDU HIV risk factors had higher odds of HCV viral testing compared to their reference groups. Shorter time since last HIV test (<6–12 months) was significantly associated with higher odds of HCV viral testing (adjusted odds ratio [aOR], 2.44; *P* < .001), whereas longer time (2 to <5 years) since first available HCV test was significantly associated with lower odds of HCV viral testing (aOR, 0.64; *P* < .0001).

**Table 3.** Relationship Between Demographic Variables and Hepatitis C Virus (HCV) Testing for the HCV/Human Immunodeficiency Virus–Coinfected Cohort for Data as of 31 December 2021

Variable	Univariable Analysis			Multivariable Analysis^a^		
	OR	95% CI	*P* Value	aOR	95% CI	*P* Value
Race						
White	Ref			Ref		
American Indian/Alaska Native	1.11	.72–1.71	.63	**0.64**	**.41–.99**	.**05**
Asian/Native Hawaiian/Pacific Islander	0.84	.51–1.37	.48	**0.51**	**.31–.84**	.**009**
Black or African American	**1.62**	**1.43–1.83**	**<**.**0001**	1.08	.932–1.241	.320
Other	1.09	.78–1.53	.62	**1.50**	**1.03–2.18**	.**037**
Ethnicity						
Non-Hispanic/Latino	Ref			Ref		
Hispanic/Latino	**0.42**	**.381–.47**	**<**.**0001**	**0.80**	**.69–.92**	.**002**
Age group, y						
<26	Ref			Ref		
26–35	1.65	.96–2.83	.07	1.71	.98–2.97	.059
36–45	1.15	.69–1.94	.59	1.50	.88–2.57	.134
46–55	1.56	.93–2.60	.091	**1.89**	**1.11–3.20**	.**019**
56–65	**1.88**	**1.13–3.13**	.**016**	**2.17**	**1.28–3.67**	.**004**
>65	1.67	.99–2.81	.057	1.76	1.02–3.03	.041
Sex						
Male	Ref			Ref		
Female	0.92	.812–1.03	.157	**0.88**	**.77–.99**	.**047**
HIV transmission						
Heterosexual contact (male-female)	Ref			Ref		
Injection drug use	**2.63**	**2.29–3.02**	**<**.**0001**	**1.34**	**1.13–1.58**	.**0006**
Male-male sexual contact	**1.96**	**1.68–2.28**	**<**.**0001**	0.96	.795–1.158	.665
Male-male sexual contact and injection drug use	**3.17**	**2.56–3.92**	**<**.**0001**	**1.51**	**1.18–1.92**	.**0009**
Other	**1.55**	**1.01–2.38**	.**043**	1.00	.63–1.59	.994
HIV suppression status (within 12 mo)						
Viral load detectable (≥200 copies/mL)	Ref			Ref		
Load undetectable (<200 copies)	**1.21**	**1.02–1.42**	.**025**	1.16	.97–1.39	.110
Time since last HIV viral load, CD4, or HIV genotype						
≤6 mo	**4.73**	**4.17–5.37**	**<**.**0001**	**2.33**	**1.95–2.79**	**<**.**0001**
>6 to 12 mo	**4.65**	**3.82–5.65**	**<**.**0001**	**2.44**	**1.93–3.09**	**<**.**0001**
>12 to 18 mo	**3.63**	**2.69–4.90**	**<**.**0001**	**1.83**	**1.32–2.54**	.**0003**
>18 mo	Ref			Ref		
Time since first available HCV^+^ result (Ab or PCR)						
<1 y	1.89	.63–5.65	.257	0.50	.16–1.56	.233
1 to <2 y	1.51	.55–4.13	.421	0.34	.12–1.00	.050
2 to <5 y	**0.79**	**.68–.91**	.**0009**	**0.64**	**.54–.76**	**<**.**0001**
≥5 y	Ref			Ref		
Time since most recent HCV (Ab or PCR) test						
<1 y	**4.64**	**3.99–5.40**	**<**.**0001**	**10.26**	**8.42–12.52**	**<**.**0001**
1 to <2 y	**8.45**	**6.33–11.28**	**<**.**0001**	**11.68**	**8.46–16.11**	**<**.**0001**
2 to <5 y	**2.52**	**2.22–2.87**	**<**.**0001**	**4.04**	**3.46–4.72**	**<**.**0001**
≥5 y	Ref			Ref		

Abbreviations: Ab, antibody; aOR, adjusted odds ratio; CI, confidence interval; HCV, hepatitis C virus; HIV, human immunodeficiency virus; OR, odds ratio; PCR, polymerase chain reaction.

^a^Adjusted for jurisdiction.

Bolded values indicate statistically significant *P*-values and 95% confidence intervals (*P* < 0.05).

### Predictors of HCV Clearance for the HIV/HCV Coinfection Cohort (Cascade Step 4)

Table 4 compares demographics of individuals who did and did not achieve HCV cure/clearance. Black/African American individuals had significantly lower odds of cure/clearance compared to White individuals (aOR, 0.83; *P* = .030). MSM HIV risk factor was associated with increased odds of cure/clearance compared to heterosexual contact (aOR, 1.46; *P* = .004). Older persons, particularly those 56–65 years old, had higher odds of cure/clearance (aOR, 2.82; *P* = .47) compared to those <26 years old. Individuals with undetectable HIV viral loads had higher odds of cure/clearance than those with detectable viral loads (aOR, 2.19; *P* < .0001). Shorter times since last HIV test (<6–12 months) and first available HCV test (<1 year) had higher odds of cure/clearance (aOR, 3.41; *P* < .0001 and aOR, 6.21; *P* < .0448, respectively). Shorter time (<1 year) since last HCV testing was associated with lower odds of cure/clearance (aOR, 0.32; *P* < .045). There were jurisdictional-level differences in achieving these outcomes (Supplementary Table 6).

**Table 4.** Relationship Between Demographic Variables and Hepatitis C Virus (HCV) Cure/Clearance for HCV/Human Immunodeficiency Virus–Coinfected Cohort for Data as of 31 December 2021

Variable	Univariable Associations			Multivariable Associations		
	OR	95% CI	*P* Value	aOR	95% CI	*P* Value
Race						
White	Ref			Ref		
American Indian/Alaska Native	**0.32**	**.16–.67**	.**002**	0.61	.29–1.29	.195
Asian/Pacific Islander	1.20	.57–2.51	.632	1.12	.52–2.41	.770
Black or African American	**1.20**	**1.02–1.40**	.**024**	**0.83**	**.69–.98**	.**030**
Other	0.68	.42–1.12	.127	0.65	.39–1.08	.098
Ethnicity						
Non-Hispanic/Latino	Ref			Ref		
Hispanic/Latino	0.94	.82–1.08	.393	0.99	.84–1.17	.903
Age group, y						
<26	Ref			Ref		
26–35	1.95	.71–5.39	.198	1.63	.57–4.66	.365
36–45	2.69	.99–7.30	.052	2.64	.94–7.41	.066
46–55	**2.93**	**1.09–7.87**	.**033**	2.73	.98–7.59	.055
56–65	**3.29**	**1.23–8.81**	.**018**	**2.82**	**1.02–7.84**	.**047**
>65	**2.99**	**1.10–8.11**	.**032**	2.27	.81–6.39	.120
Sex						
Male	Ref			Ref		
Female	1.03	.88–1.20	.759	0.87	.74–1.03	.097
Transmission						
Heterosexual contact (male-female)	Ref			Ref		
Injection drug use	**1.31**	**1.06–1.62**	.**012**	1.10	.88–1.38	.418
Male-male sexual contact	1.25	.99–1.58	.058	**1.46**	**1.13–1.89**	.**004**
Male-male sexual contact and injection drug use	0.95	.72–1.25	.693	1.22	.90–1.65	.204
Other	1.04	.56–1.94	.896	1.18	.61–2.27	.619
HIV suppression status (within 12 mo)						
Viral load detectable (≥200 copies/mL)	Ref			Ref		
Load undetectable (<200 copies)	**2.21**	**1.74–2.79**	**<**.**0001**	**2.19**	**1.71–2.81**	**<**.**0001**
Time since last HIV viral load, CD4, or HIV genotype						
≤6 mo	**4.08**	**3.26–5.11**	**<**.**0001**	**4.99**	**3.67–6.79**	**<**.**0001**
>6–12 mo	**2.26**	**1.71–2.98**	**<**.**0001**	**3.41**	**2.40–4.86**	**<**.**0001**
>12–18 mo	**1.94**	**1.28–2.95**	.**0020**	**2.35**	**1.46–3.79**	.**0005**
>18 mo	Ref			Ref		
Time since most recent HCV (Ab or PCR) test						
<1 y	0.79	.26–2.36	.671	**0.32**	**.10–.97**	.**045**
1 to <2 y	2.84	.97–8.32	.057	1.14	.37–3.44	.823
2 to <5 y	**1.35**	**1.10–1.65**	.**004**	1.12	.90–1.39	.322
≥5 y	Ref			Ref		
Time since first available HCV^+^ result (Ab or PCR)						
<1 y	**7.29**	**5.96–8.91**	**<**.**0001**	**6.21**	**5.00–7.72**	**<**.**0001**
1 to <2 y	**8.19**	**6.29–10.67**	**<**.**0001**	**6.41**	**4.87–8.44**	**<**.**0001**
2 to <5 y	**3.74**	**3.06–4.57**	**<**.**0001**	**3.35**	**2.72–4.12**	**<**.**0001**
≥5 y	Ref			Ref		

Abbreviations: Ab, antibody; aOR, adjusted odds ratio; CI, confidence interval; HCV, hepatitis C virus; HIV, human immunodeficiency virus; OR, odds ratio; PCR, polymerase chain reaction.

Bolded values indicate statistically significant *P*-values and 95% confidence intervals (*P* < .05).

## DISCUSSION

Using the CDC's HCV surveillance-based clearance cascade methodology to examine HCV care continuum milestones for people coinfected with HIV, we found that HCV clearance rates increased from 31.6% to 42.4% among 6 jurisdictions over 2 years. Older persons, those with MSM as HIV risk factor, and those with HIV viral suppression were more likely to achieve clearance. Given the higher likelihood of adverse health outcomes for coinfected individuals, these findings highlight the need for continued efforts to address this population.

DAA treatment leads to reduced mortality. In a Veterans Affairs cohort, persons with HCV without advanced liver disease who achieved sustained virologic response (SVR) had reduced mortality compared to those treated without SVR (hazard ratio [HR], 0.44) and untreated persons (HR, 0.32) [18]. For persons with HIV/HCV coinfection, Breskin et al analyzed data from the Women's Interagency HIV Study and Multicenter AIDS Cohort Study, revealing a 4.3% mortality risk difference between people with HIV with or without HCV over 10 years, showing the effectiveness of DAAs in reducing mortality [19]. Despite successful HCV treatment, persons with HIV/HCV, particularly those with advanced liver disease, remain at increased mortality risk, highlighting the benefits of early diagnosis and DAA treatment [20]. Additionally, increased mortality in coinfected patients due to non-liver-related deaths in the DAA era shows the ongoing need for clinical monitoring in this group [21, 22].

Our study uses the HCV clearance cascade to examine public health trends in achieving HCV cure/clearance for coinfected persons. We found that, compared to HCV-monoinfected persons, the coinfected group had more recent HCV testing, indicating more recent engagement in care. The overall clearance rate of 42.4% in 6 jurisdictions compares favorably to the 34% clearance among a monoinfected group published by Wester et al [11], suggesting higher engagement in HCV care for those with coinfection. We found that persons with HIV viral suppression were twice as likely to achieve HCV cure. We speculate that enhanced efforts to better understand and promote engagement in HIV care contribute to this outcome. Building capacity within HIV clinics to promote HCV cure/clearance has been a focus of Ryan White HIV programs through investments in training platforms (eg, national HCV training modules) and care models [23]. The role of multidisciplinary care with wraparound services has been integral to HIV care as demonstrated by successes in Ryan White–funded programs, which demonstrate higher retention and viral suppression rates compared to national averages [24]. Various reengagement strategies for persons out of HIV care have been piloted, with 1 meta-analysis showing reengagement interventions increasing patient return to care by 20% compared to the standard of care [25]. A systematic review showed that interventions focused on utilizing surveillance data together with clinic-level resources and staff had better reengagement outcomes compared to interventions focused on field workers or healthcare personnel [26].

Creating the HCV clearance cascade, a key strategy within the CDC's Division of Viral Hepatitis 2025 Strategic Plan, enables analysis of strengths and gaps, which can guide future implementation strategies [9]. Creating jurisdictional clearance cascades requires enhancements in data collection and matching, along with enhancing HCV surveillance to gather longitudinal laboratory disposition data. We created a Jurisdictional Data Collection Tool, enabling longitudinal data collection and cascade generation based on standardized definitions, illustrating longitudinal changes at each cascade step. In this sample of 6 jurisdictions, driven by changes in the denominator (eg, excluding the deceased or relocated individuals between reporting quarters), viral testing rates increased by 2.1% and cure/clearance rates increased by 10.8%. Despite these modest increases, final rates for both measures remain below the US National Viral Hepatitis Strategy and the World Health Organization's 2030 target for HCV elimination. To address similar viral testing gaps nationwide, the CDC and the Association of Public Health Laboratories have issued updated guidance recommending automatic reflex PCR testing on specimens with reactive antibody results [27, 28]. This approach has shown to increase viral testing by removing barriers such as the need for additional follow-up HCV testing visits [27]. While laboratories may need to adjust workflows to implement this new guidance, New York State has already adopted this practice, demonstrating its feasibility [28, 29]. To address gaps in cure/clearance rates, public health departments can assist clinics in reengagement efforts for patients falling into cascade gaps. The HCV clearance cascade created using public health surveillance can help streamline outreach efforts [30, 31]. Health departments can use D2C methods such as sharing lists of persons needing HCV viral testing and treatment with clinics who could then directly reach out to patients. An analysis of demographic and clinical characteristics of patients who fall into testing and treatment gaps could help inform targeted outreach.

Customizing a standard approach for cascade creation for multiple public health jurisdictions presented challenges. For example, Puerto Rico lacked a fully developed HCV surveillance system and used CAREWare, a system not designed for disease reporting, resulting in a low viral testing rate of 27.7%. In contrast, FLOC’s 88.4% testing rate likely reflects inclusion of pre-2016 cases (prior to the change in the CDC/Council of State and Territorial Epidemiologists case definition requiring PCR-positive results for confirmation), possibly leading to an inflated viral testing rate. Other major challenges include suboptimal data capacity of HCV surveillance databases compared to eHARS, the HIV database. HCV surveillance data lacked race and ethnicity details (Supplementary Table 4*A* and 4*B*) as well as residence information. Limited electronic laboratory reporting into HCV data repositories has led to a significant backlog needing manual data entry, which is prone to errors and incompleteness [31]. Some jurisdictions were not equipped to collect HCV PCR–negative results, which are required to assess cure/clearance. Finally, many jurisdictions were not legally required to report HCV antibody–negative results, making it impossible to characterize the upstream part of the cascade, namely, HCV testing.

Another challenge was the availability of trained HCV surveillance staff. High staff turnover within HCV programs hindered the development of sustainable expertise. HIV programs were substantially better staffed than HCV programs, with the median number of 4 FTEs compared to a median of 1.25 for HCV—more than 3 times higher (Supplementary Table 2). When standardized by disease burden, the disparity was even more striking: HIV programs had a median of 0.37 FTEs per 1000 people with HIV, while HCV programs had only 0.023 FTEs per 1000 people with HCV—a 16-fold difference (Supplementary Table 2). Notably, FLOC had the highest HCV staffing rate and also demonstrated the highest odds of both HCV viral testing and cure/clearance among all jurisdictions, suggesting that increased staffing capacity may contribute to improved surveillance and care outcomes (Supplementary Table 6). We also observed silos between HIV and HCV programs within health departments, varying by jurisdiction and stemming from inherent policies protecting patient data as well as inadequate interprogram collaboration and communication. Such silos led to inefficiencies, data quality issues, and hampered project goals. Jurisdictions who prioritize HCV outcomes can enhance their success by developing protocols that integrate and promote collaboration, emphasizing routine data sharing between HIV and HCV programs. To enable sustainability of clearance cascade efforts, jurisdictions need dedicated time and resources for training appropriate staff and maintaining the infrastructure to serially follow progress. Most jurisdictions do have the precedent of routinely creating and posting their HIV care cascades, so adding HCV clearance cascades to their routine reporting would theoretically be possible.

Our study had several limitations. Aggregate data limited individual-level granularity, preventing analysis of causal relationships, particularly for most impactful variables on viral testing and clearance outcomes. The use of HCV surveillance data was often incomplete (particularly for PCR result reporting) and likely led to an underestimation of cure/clearance rates at the jurisdiction level. Moreover, the cascades did not incorporate HCV treatment data (eg, DAA prescriptions), limiting our ability to determine whether individuals without documented cure/clearance were currently undergoing treatment or had not yet initiated it. Additionally, the HCV clearance cascade cannot determine if those not cured/cleared are currently in treatment or have been cured but missed follow-up evaluation. As an alternative, clinic-based cascades may offer more complete individual-level outcome data, including treatment initiation and completion, but they may be more labor-intensive for clinics to implement and require more resources [32]. Last, this study was conducted during the coronavirus disease 2019 pandemic, which often diverted staff time to address pandemic-related work.

## CONCLUSIONS

We adapted the CDC standardized surveillance-based HCV clearance cascade for persons with HIV/HCV coinfection and demonstrated increases in viral clearance over 2 years for a diverse group of health department jurisdictions. While viral clearance rates above 40% in this coinfected group surpass those for monoinfected persons, these still fall short of the 80% goal set by national strategic guidelines. Given the poorer health outcomes among persons with coinfection, public health strategies that build on engagement in HIV care through established clinical models and public health infrastructure can be leveraged to improve HCV outcomes.

## Supplementary Material

ofaf412_Supplementary_Data

## Supplementary Data

Supplementary materials are available at *Open Forum Infectious Diseases*  online. Consisting of data provided by the authors to benefit the reader, the posted materials are not copyedited and are the sole responsibility of the authors, so questions or comments should be addressed to the corresponding author.
