# Supplementary material for: Hepatitis C Virus (HCV) Clearance Cascade for Persons With Human Immunodeficiency Virus (HIV)/HCV Coinfection Using Health Department Surveillance Data Among 7 US Jurisdictions Highlights the Role of HIV Care Engagement
Source: Open Forum Infect Dis. 2025 Aug 7;12(8):ofaf412. doi: 10.1093/ofid/ofaf412 (PMC12342353; doi:10.1093/ofid/ofaf412)
Supplement: ofaf412_Supplementary_Data [file ofaf412_supplementary_data.docx]

**Supplemental Table 1:** Minimum Required Tasks Needed to Create and Publish the HCV Clearance Cascade for HIV/HCV Co-infected People

| 1. Has HIV database | 5. HCV database collects HCV PCR positives results | 9. Valid matching tool/program available | 13. Able to identify out of jurisdiction persons | 17. Use HCV results to create dispositions for cascade placement |
| --- | --- | --- | --- | --- |
| 2. HIV database demographics are updated | 6. HCV PCR positive results are current | 10. Experience using matching tool | 14. Ability to identify people needing HCV PCR testing | 18. Has created an HCV cascade |
| 3. Has HCV database | 7. HCV database collects HCV PCR negative results | 11. Ability to create co-infected list by matching HIV and HCV | 15. Ability to identify people who self-cleared HCV | 19. Has published/shared completed cascade |
| 4. HCV database collects HCV antibody positive result | 8. HCV PCR negative results are current | 12. Able to identify deceased persons | 16. Ability to identify people needing HCV treatment |  |

**Supplemental Table 2:** Jurisdictional HIV and HCV Surveillance Details

| Jurisdiction | HIV surveillance program FTE^a^ | HCV surveillance program FTE^b^ | FTE per 1,000 with HIV^c^ | FTE per 1,000 with HCV^d^ | eHARS start year | HCV database | HCV database start year | Software used for database matching |  |
| --- | --- | --- | --- | --- | --- | --- | --- | --- | --- |
| AZ | 10 | 2 | 0.54 | 0.014 | 1981 | MEDSIS | 1998 | Match*Pro |  |
| CT | 4 | 1.25 | 0.37 | 0.016 | 1981 | CTEDSS | 1994 | SAS |  |
| FLOC | 1^e^ | 1 | 0.11 | 0.069 | 1979 | MERLIN | 2002 | SAS |  |
| KY | 6 | 2.5 | 0.71 | 0.030 | 1982 | NNDSS | 2013 | Match*Pro |  |
| MI | 3.5 | 9.6 | 0.21 | 0.063 | 1986 | MDSS | 2004 | SAS |  |
| NV | 2 | 0.35 | 0.17 | 0.007 | 1998 | NBS | 2015 | Match*Pro |  |
| PR | 5 | 1 | 0.52 | NA | 1987 | CAREWare | 2019 | SAS |  |
| *Abbreviations:* MEDSIS, Medical Electronic Disease Surveillance and Intelligence System; CTEDSS, Connecticut Electronic Disease Surveillance System; NNDSS, National Notifiable Disease Surveillance System; MDSS, Michigan Disease Surveillance System; eHARS electronic HIV AIDS reporting system; FTE, Full-Time-Equivalent  *a. Total FTE for HIV surveillance programs is 31.5; median FTE is 4*  *b. Total FTE for HCV surveillance programs is 17.7; median FTE is 1.25*  *c. Number of persons with HIV per jurisdiction can be found in Supplemental Table 4a*  *d. Number of persons with HCV per jurisdiction can be found in Supplemental Table 4b*  *e. Indicates state level staff supporting local public health department* | | | | | | | | | |

**Supplemental Table 3: Jurisdictional Cascade Creation Methodology by Health Department Jurisdiction**

| **Jurisdiction** | **Matching** | | | **Cascade Creation** | |
| --- | --- | --- | --- | --- | --- |
|  | **Steps** | **Person(s) Responsible** | **Software** | **Steps, Disposition Creation** | **Person(s) Responsible** |
| AZ | 1.create PWH list using eHARS 2.create HCV list using ELR data 3. match list based on identifiable variables using Match*Pro (first name, last name, DOB, sex assigned at birth) | PWH list creation = HIV epidemiologist  HCV list creation = HCV epidemiologist  Matching = HIV epidemiologist | SAS | ELR LOINC and SNOMED codes were used to standardized HCV labs into reactive and non-reactive & HCV Ab, HCV RNA and HCV Genotype tests. Observations were then placed into the disposition categories utilized in the cascade model | HCV epidemiologist |
| CT | Step 1: Create the PWH list using eHARS  Step 2: Create HCV list using CTEDSS   Step 3: Match the list based on the following identifiable variables using SAS (First name, last name, DOB, SSN, and Soundex) | PWH list creation = HIV/HCV surveillance epidemiologist    HCV list create = HIV/HCV surveillance epidemiologist   Matching done by HIV/HCV surveillance epidemiologist | SAS | Before the cascade is created, CT DPH generates an updated disposition for the cohort using SAS code that links the latest HCV and HIV lab information available for any patient selected for this project. CT DPH then packages the results in excel and shares with Yale University. Yale in turn creates the cascade and shares it with CT DPH. CT DPH reviews the cascade with clinics. CT DPH uses the SAS code to determine the disposition of the person’s HCV status, based on what’s available in CT DPH database. The data goes back from 1994 to present, where a patient's historical disease evolution is recorded | HIV/HCV surveillance epidemiologist |
| FLOC | 1.Match HCV database w/eHARS based on identifiable variables using LinkPlus and CLEAR for manual validation of fuzzy matches (first name, last  name, DOB, SSN, Alias names, DOB & SSN, client address 2.Import the MERLIN hepatitis ID number and HCV related data from matched cases into eHARS  3.Create PWH for HIV and HIV/HCV dataset from eHARS using SAS 4.Create HCV care database by matching HIV/HCV dataset w/MERLIN using the MERLIN hepatitis ID number contained in the HIV/HCV dataset to obtain most recent HCV-related care data | HCV care list creation:  State HCV surveillance epidemiologist PWH, HIV/HCV list creation: State DPH HIV epidemiologist. Create HCV viral clearance cascade: collaboration of state DPH HIV and HCV epidemiologist | SAS | the SAS code gathered labs from Merlin with a hepatitis related ICD-9 code (i.e., 07051, 07054, 07058), checked the collection of labs for each case and determined which group the case fell into (e.g., AB+ but no PCR, PCR+ then PCR-), and then information was matched back to the HIV dataset based on the state (HIV case state number) | collaboration of State DPH HIV and HCV epidemiologists |
| MI | 1.Create the PWH list using eHARS  2.Create HCV list using MDSS 2a. a systematic manual review of all HCV cases was necessary due to surveillance system and case classification changes and constraints. 2a(i)MDSS has evolved; merged data to new versions and entered data retrospectively among other modifications. Cases classified as confirmed or probable were considered persons testing positive for HCV even if the test was not documented (to satisfy case classification rules, a positive test had to have been present in some form at time of referral. 2b.Upon manual review, each HCV case was classified in a dichotomous fashion as "ever infected" or "never infected"   3.Match the list based on identifiable variables (first name, last name, middle name, DOB) | PWH list creation = HIV epidemiologist  HCV list creation = HCV epidemiologist  Matching = HIV epidemiologist | SAS | A stepwise process to interpret laboratory tests and results through:   1.Delimiting string values of test names and corresponding results   2.Formatting, cleaning, and standardizing test name and result values   3.Query test names and results for key words to automate identification of laboratory results   3a.Some specific combinations of results are flagged for manual review as identified in manual quality assurance checks   4.Transpose the sequence of lab hepatitis C lab results for each person into a single, sequential, string value in order to query for seroconversions by using key words   5.Utilize analysis of seroconversion sequences (or lack thereof) to categorize each case into a disposition on the cascade Sequences of lab results were used to classify each case based on lab result and order. Amount of time between RNA results with regard to seroconversion was not assessed. | HCV epidemiologist |
| NV | Step 1: Create the PWH list using eHARS and CAREWare   Step 2: Create HCV list using Trisano and NBS  Step 3: Match the list based on the following identifiable variables using SAS Match*Pro: First name, Last Name, DOB, Birth sex | PWH list creation = HIV epidemiologist    HCV list crate = HCV epidemiologist (Clark County data); NDBPH (rest of counties)    Matching done by HCV epidemiologist | SAS | Determine patient’s lab history and results for appropriate time period for the quarter; 2. Based on lab history and results, assign appropriate disposition from SHILD rules for current quarter; 3.  With each advancing quarter, algorithm looks at all previous labs and either keeps patient in previous disposition (if no new labs or new labs still keep patient in current disposition based on SHILD rules), or moves patient to a different disposition based on SHILD rules. | HCV epidemiologist |
| PR | Step 1: Create the PWH list using eHARS   Step 2: Create HCV list using NBS or CAREWare or MS Access   Step 3: Match the list based on the following identifiable variables using SAS:  First name, Last Name, DOB, SSN, Patient ID | Matching done by HIV epidemiologist and HCV coordinator | SAS or R | The patient result set and the dates of the results were compared to determine to which categories it applied. | HCV surveillance coordinator |

**Supplemental Table 4a: HIV Demographic and Clinical Characteristics of Participating Jurisdictions**

| HIV Demographic variable | AZ  (N=18,402) | CT  (N=10,705) | FLOC  (N=9,273) | KY  (N=8,450) | MI  (N=16,913) | NV  (N=11,654) | PR  (N=9,558) |
| --- | --- | --- | --- | --- | --- | --- | --- |
| **Race** | | | | | | | |
| White / Caucasian | 78.0% | 63.3% | 52.0% | 62.3% | 38.2% | 63.2% | 83.7% |
| Black or African American | 14.4% | 34.1% | 46.7% | 36.1% | 57.1% | 29.6% | 10.8% |
| Asian | 1.6% | 1.1% | 0.9% | 1.1% | 1.0% | 3.7% | 0.1% |
| Native Hawaiian or Pacific Islander | 0.0% | 0.1% | 0.2% | 0.1% | 0.0% | 0.7% | 0.0% |
| American Indian / Alaska Native | 4.2% | 0.1% | 0.2% | 0.4% | 0.3% | 0.7% | 0.0% |
| Other | 1.8% | 1.3% | 0.0% | 0.0% | 3.4% | 2.1% | 5.4% |
| Unknown / Missing | 0.0% | 0.0% | 4.6% | 1.4% | 1.5% | 4.2% | 4.7% |
| **Ethnicity** | | | | | | | |
| Hispanic / Latino | 30.1% | 33.6% | 27.2% | 7.5% | 6.7% | 25.3% | 97.2% |
| Non-Hispanic / Latino | 69.9% | 66.4% | 72.8% | 92.5% | 93.3% | 74.7% | 2.8% |
| Unknown / Missing | 0.0% | 0.0% | 0.3% | 1.3% | 5.9% | 0.8% | 0.0% |
| **Current Age** | | | | | | | |
| Mean | 48 | 52 | 47 | 48 | 46 | 47 | 52 |
| SD | 13 | 13 | 13 | 13 | 14 | 13 | 13 |
| **Age Groups** | | | | | | | |
| <18 | 0.5% | 0.2% | 0.4% | 0.4% | 0.6% | 0.2% | 0.5% |
| 18-25 | 3.7% | 2.5% | 4.6% | 3.2% | 5.3% | 3.7% | 1.8% |
| 26-35 | 16.4% | 10.6% | 18.0% | 17.9% | 20.9% | 20.5% | 10.1% |
| 36-45 | 19.4% | 14.5% | 19.8% | 21.0% | 18.4% | 20.6% | 16.0% |
| 46-55 | 27.5% | 28.7% | 27.0% | 25.9% | 25.8% | 26.8% | 29.5% |
| 56-65 | 23.7% | 31.1% | 22.0% | 23.6% | 21.8% | 20.6% | 29.8% |
| 66-75 | 7.4% | 10.6% | 6.9% | 7.0% | 6.2% | 6.4% | 10.0% |
| over 75 years | 1.4% | 1.8% | 1.3% | 1.0% | 1.0% | 1.2% | 2.3% |
| Unknown / Missing | 0.0% | 0.0% | 0.0% | 0.0% | 0.0% | 1.1% | 0.0% |
| **Sex at Birth** | | | | | | | |
| Male | 85.7% | 66.6% | 76.0% | 80.8% | 79.3% | 84.3% | 67.3% |
| Female | 14.3% | 33.4% | 24.0% | 19.2% | 20.7% | 15.7% | 32.7% |
| Unknown/Missing | 0.0% | 0.0% | 0.0% | 0.0% | 0.00% | 0.0% | 0.0% |
| **HIV Transmission** | | | | | | | |
| Male-male sexual contact | 68.2% | 35.7% | 57.1% | 55.1% | 64.7% | 72.1% | 20.7% |
| Injection drug use | 9.3% | 28.6% | 8.0% | 9.1% | 6.5% | 7.1% | 11.8% |
| Male-male sexual contact and injection drug use | 8.8% | 2.6% | 3.1% | 5.9% | 4.5% | 6.9% | 0.4% |
| Heterosexual contact (male-female) | 12.3% | 30.9% | 30.0% | 18.7% | 22.4% | 13.0% | 64.2% |
| Other | 1.4% | 2.2% | 1.8% | 11.2% | 1.9% | 0.9% | 2.9% |
| Unknown / Missing | 9.8% | 10.9% | 0.0% | 0.0% | 14.3% | 11.2% | 3.0% |
| **HIV Suppression Status (within 12 months)** | | | | | | | |
| Viral Load Undetectable (<200 copies) | 90.3% | 92.2% | 88.7% | 62.4% | 89.3% | 89.0% | 89.4% |
| Viral Load Detectable (>=200 copies) | 9.7% | 7.8% | 11.3% | 37.6% | 10.7% | 11.0% | 10.6% |
| Unknown / Missing | 38.0% | 20.1% | 22.8% | 9.7% | 18.2% | 34.6% | 36.9% |
| **Time Since Last HIV Viral Load, CD4, or HIV Genotype** | | | | | | | |
| ≤6 months | 54.7% | 73.5% | 69.9% | 63.1% | 74.8% | 69.5% | 0.0% |
| > 6 - 12 months | 24.2% | 10.8% | 11.0% | 12.1% | 12.3% | 11.7% | 100.0% |
| >12 - 18 months | 4.8% | 3.0% | 4.3% | 3.0% | 3.7% | 5.1% | 0.0% |
| >18 months | 16.3% | 12.7% | 14.8% | 21.8% | 9.2% | 13.7% | 0.0% |
| Unknown / Missing | 14.4% | 3.6% | 5.2% | 5.2% | 2.5% | 13.3% | 36.9% |
| *Notes:*   - Percentages exclude unknowns from the denominator - Percentages for unknowns are calculated separately | | | | | | | |

**Supplemental Table 4b: HCV Demographic and Clinical Characteristics of Participating Jurisdictions**

| HCV Demographic Variable | AZ (N=143,996) | CT (N=78,037) | FLOC (N=14,503) | KY (N=82,365) | MI (N=151,880) | NV (N=49,176) | PR (N=NA) |
| --- | --- | --- | --- | --- | --- | --- | --- |
| **Race** | | | | | | | |
| White / Caucasian | 83.3% | 74.9% | 68.2% | 89.5% | 64.7% | 68.3% | NA |
| Black or African American | 6.8% | 22.2% | 15.1% | 3.0% | 29.6% | 20.8% | NA |
| Asian | 0.5% | 1.5% | 2.3% | 0.3% | 0.8% | 2.2% | NA |
| Native Hawaiian or Pacific Islander | 0.0% | 0.0% | 0.0% | 0.0% | 0.1% | 0.7% | NA |
| American Indian / Alaska Native | 7.7% | 0.3% | 0.3% | 0.3% | 1.0% | 0.9% | NA |
| Other | 1.7% | 1.1% | 14.1% | 6.9% | 3.8% | 7.1% | NA |
| Unknown / Missing | 72.1% | 65.2% | 29.1% | 76.1% | 27.5% | 81.4% | NA |
| **Ethnicity** | | | | | | | |
| Hispanic / Latino | 23.2% | 21.9% | 25.3% | 0.8% | 4.1% | 15.2% | NA |
| Non-Hispanic / Latino | 76.8% | 78.1% | 74.7% | 99.2% | 95.9% | 84.8% | NA |
| Unknown / Missing | 76.8% | 55.4% | 48.8% | 86.0% | 55.2% | 83.5% | NA |
| **Current Age** | | | | | | | |
| Mean | 46 | 55 | 47 | 46 | 48 | 60 | NA |
| SD | 14 | 14 | 14 | 14 | 15 | 15 | NA |
| **Age** | | | | | | | |
| <18 | 0.7% | 0.5% | 0.5% | 0.5% | 1.0% | 0.4% | NA |
| 18-25 | 6.1% | 1.7% | 6.8% | 1.4% | 7.0% | 1.1% | NA |
| 26-35 | 16.5% | 10.4% | 18.7% | 23.1% | 13.7% | 6.6% | NA |
| 36-45 | 23.6% | 13.2% | 18.5% | 28.9% | 15.9% | 8.9% | NA |
| 46-55 | 29.5% | 19.7% | 24.8% | 17.5% | 29.1% | 12.9% | NA |
| 56-65 | 16.6% | 33.1% | 23.2% | 18.8% | 24.2% | 32.5% | NA |
| 66-75 | 5.3% | 17.5% | 5.9% | 8.7% | 6.7% | 27.8% | NA |
| over 75 years | 1.7% | 3.9% | 1.6% | 1.1% | 2.4% | 9.8% | NA |
| Unknown / Missing | 0.6% | 18.8% | 0.1% | 0.1% | 0.6% | 0.5% | NA |
| **Sex at Birth** | | | | | | | |
| Male | 65.0% | 63.6% | 66.4% | 55.6% | 61.0% | 59.9% | NA |
| Female | 35.0% | 36.4% | 33.6% | 44.4% | 39.0% | 40.1% | NA |
| Unknown/Missing | 0.3% | 0.1% | 0.2% | 0.3% | 0.6% | 4.6% | NA |
| **Time Since First Available HCV+ Result (Ab or PCR)** | | | | | | | |
| <1 year | 6.0% | 4.2% | NA | 12.5% | 39.0% | 8.7% | NA |
| 1 - <2 years | 5.2% | 5.4% | NA | 16.4% | 12.5% | 8.8% | NA |
| 2 - <5 years | 14.2% | 12.6% | NA | 45.9% | 25.5% | 21.3% | NA |
| ≥ 5 years | 74.6% | 77.8% | NA | 25.2% | 23.0% | 61.2% | NA |
| Unknown / Missing | 0.0% | 0.0% | NA | 0.0% | 60.7% | 0.2% | NA |
| **Time Since Most Recent HCV (Ab or PCR) test** | | | | | | | |
| <1 year | 1.4% | 18.1% | 0.0% | 34.9% | 50.3% | 18.3% | NA |
| 1 - <2 years | 3.1% | 11.5% | 0.0% | 21.9% | 9.2% | 12.9% | NA |
| 2 - <5 years | 13.2% | 15.5% | 0.0% | 32.0% | 21.0% | 24.6% | NA |
| ≥ 5 years | 82.3% | 54.9% | 0.0% | 11.2% | 19.5% | 44.2% | NA |
| Unknown / Missing | 0.0% | 0 | 100.00% | 0.0% | 59.3% | 0.1% | NA |
| *Notes:*   - Percentages exclude unknowns from the denominator - Percentages for unknowns are calculated separately | | | | | | | |

**Supplemental Table 4c: HIV/HCV Co-infected Demographic and Clinical Characteristics of Participating Jurisdictions**

| Co-infected Demographic Variable | AZ (N=1,500) | CT (N=2,220) | FLOC (N=472) | KY  (N=596) | MI  (N=1,060) | NV  (N=779) | PR (N=1,205) |
| --- | --- | --- | --- | --- | --- | --- | --- |
| **Race** | | | | | | | |
| White / Caucasian | 72.8% | 44.3% | 57.9% | 66.0% | 41.4% | 65.5% | 81.0% |
| Black or African American | 15.4% | 53.1% | 42.1% | 34.0% | 57.7% | 28.1% | 13.9% |
| Asian | 1.5% | 0.5% | 0.0% | 0.0% | 0.9% | 3.4% | 0.0% |
| Native Hawaiian or Pacific Islander | 0.0% | 0.0% | 0.0% | 0.0% | 0.0% | 0.7% | 0.0% |
| American Indian / Alaska Native | 7.6% | 0.0% | 0.0% | 0.0% | 0.0% | 0.9% | 0.0% |
| Other | 2.7% | 2.1% | 0.0% | 0.0% | 3.4% | 1.4% | 5.1% |
| Unknown / Missing | 0.0% | 0.0% | 4.9% | 0.0% | 0.7% | 2.2% | 0.4% |
| **Ethnicity** | | | | | | | |
| Hispanic / Latino | 24.7% | 41.5% | 31.8% | 3.0% | 5.6% | 19.1% | 98.8% |
| Non-Hispanic / Latino | 75.3% | 58.5% | 68.2% | 97.0% | 94.4% | 80.9% | 1.2% |
| Unknown / Missing | 0.0% | 0.0% | 0.0% | 0.0% | 3.2% | 1.3% | 0.0% |
| **Current Age** | | | | | | | |
| Mean | 52 | 57 | 52 | 48 | 51 | 53 | 54 |
| SD | 11 | 9 | 12 | 13 | 13 | 12 | 10 |
| **Age Groups** | | | | | | | |
| <18 | 0.0% | 0.0% | 0.0% | 0.0% | 0.0% | 0.0% | 0.0% |
| 18-25 | 1.0% | 0.4% | 0.0% | 2.2% | 2.3% | 1.2% | 0.0% |
| 26-35 | 7.5% | 2.8% | 13.1% | 20.6% | 14.2% | 11.4% | 2.8% |
| 36-45 | 17.6% | 7.3% | 17.2% | 20.4% | 15.0% | 17.6% | 17.8% |
| 46-55 | 30.6% | 26.9% | 26.0% | 19.9% | 25.7% | 27.3% | 31.4% |
| 56-65 | 34.5% | 46.3% | 31.6% | 29.5% | 30.3% | 32.5% | 37.3% |
| 66-75 | 8.1% | 15.0% | 12.0% | 7.4% | 11.9% | 9.0% | 10.1% |
| over 75 years | 0.7% | 1.3% | 0.0% | 0.0% | 0.6% | 1.0% | 0.6% |
| Unknown / Missing | 0.0% | 0.0% | 0.0% | 0.0% | 0.0% | 0.0% | 0.0% |
| **Sex at Birth** | | | | | | | |
| Male | 83.7% | 67.8% | 73.1% | 73.0% | 77.5% | 82.7% | 72.9% |
| Female | 16.3% | 32.2% | 26.9% | 27.0% | 22.5% | 17.3% | 27.1% |
| Unknown/Missing | 0.0% | 0.0% | 0.0% | 0.0% | 0.0% | 0.0% | 0.0% |
| **HIV Transmission Type** | | | | | | | |
| Male-male sexual contact | 37.9% | 9.7% | 39.1% | 25.0% | 38.3% | 45.1% | 9.3% |
| Injection drug use | 31.2% | 72.4% | 30.0% | 42.7% | 32.3% | 27.2% | 34.6% |
| Male-male sexual contact and injection drug use | 22.4% | 4.4% | 9.4% | 14.7% | 13.0% | 19.1% | 1.3% |
| Heterosexual contact (male-female) | 6.8% | 12.7% | 21.5% | 9.5% | 13.1% | 7.5% | 53.1% |
| Other | 1.7% | 0.8% | 0.0% | 8.2% | 3.3% | 1.1% | 1.7% |
| Unknown / Missing | 6.3% | 4.6% | 0.0% | 0.0% | 8.8% | 6.4% | 0.6% |
| **HIV Suppression Status (within 12 months)** | | | | | | | |
| Viral Load Undetectable (<200 copies) | 87.6% | 91.7% | 84.6% | 68.4% | 87.1% | 86.3% | 86.6% |
| Viral Load Detectable  (>=200 copies) | 12.4% | 8.3% | 15.4% | 31.6% | 12.9% | 13.7% | 13.4% |
| Unknown / Missing | 27.0% | 13.0% | 18.6% | 3.0% | 14.2% | 23.1% | 17.5% |
| **Time Since Last HIV Viral Load, CD4, or HIV Genotype** | | | | | | | |
| ≤6 months | 59.0% | 80.3% | 71.5% | 73.8% | 77.6% | 73.8% | 88.1% |
| >6 - 12 months | 22.4% | 10.5% | 11.6% | 12.1% | 12.4% | 9.9% | 11.9% |
| >12 - 18 months | 4.7% | 1.9% | 3.2% | 3.9% | 3.4% | 5.4% | 0.0% |
| >18 months | 13.9% | 7.3% | 13.7% | 10.2% | 6.6% | 10.9% | 0.0% |
| Unknown / Missing | 7.5% | 2.6% | 1.1% | 2.0% | 1.5% | 3.2% | 17.4% |
| **Time Since First Available HCV+ Result (Ab or PCR)** | | | | | | | |
| <1 year | 7.5% | 2.4% | 6.4% | 15.8% | 12.4% | 9.8% | NA |
| 1 - <2 years | 5.7% | 2.9% | 7.4% | 18.0% | 10.5% | 13.1% | NA |
| 2 - <5 years | 19.0% | 6.4% | 27.1% | 35.8% | 40.5% | 29.5% | NA |
| ≥ 5 years | 67.8% | 88.3% | 59.1% | 30.3% | 36.6% | 47.6% | NA |
| Unknown / Missing | 0.0% | 0.0% | 7.8% | 0.0% | 24.0% | 0.0% | NA |
| **Time Since Most Recent HCV (Ab or PCR) Test** | | | | | | | |
| <1 year | 12.9% | 37.3% | 48.7% | 56.5% | 29.0% | 32.2% | 46.2% |
| 1 - <2 years | 14.9% | 16.6% | 19.6% | 17.8% | 17.5% | 19.5% | 10.0% |
| 2 - <5 years | 23.5% | 15.5% | 20.5% | 17.8% | 36.7% | 30.4% | 43.8% |
| ≥ 5 years | 48.7% | 30.6% | 11.2% | 7.8% | 16.8% | 17.8% | 0.0% |
| Unknown / Missing | 0.0% | 0.0% | 7.2% | 0.0% | 17.9% | 0.0% | 0.0% |
| *Notes:*   - Percentages exclude unknowns from the denominator - Percentages for unknowns are calculated separately | | | | | | | |

**Supplemental Table 5: HCV Clearance Cascade Steps as of Quarter 4 by Jurisdiction (6 jurisdictions)**

| Jurisdiction | 1. Ever Infected  N | 2.Viral Testing  N (%) | 3.Initial Infection N (%) | 4.Cured/Cleared  N (%) | 5. Persistent  N (%) |
| --- | --- | --- | --- | --- | --- |
| AZ | 1381 | 958 (69.4%) | 849 (88.6%) | 180 (21.2%) | 7 (3.9%) |
| CT | 2083 | 1645(79.0%) | 1328 (80.7%) | 708 (53.3%) | 0 (0.0%) |
| FLOC | 345 | 305 (88.4%) | 260 (85.2%) | 164 (63.1%) | 0 (0.0%) |
| MI | 940 | 613 (65.2%) | 511(83.4%) | 247 (48.3%) | 11 (4.5%) |
| NV | 672 | 458 (68.2%) | 372 (81.2%) | 152 (40.9%) | 3 (2.0%) |
| PR | 1141 | 316 (27.7%) | 246 (77.8%) | 60 (24.4%) | 4 (6.7%) |

**Supplemental Table 6: Relationship between jurisdictions for achieving HCV viral testing and HCV cure/clearance: (data as of 12/31/2021)**

| Jurisdiction | Viral Testing vs No Viral Testing (Step 2)^1^ | | Cured/Cleared vs Not Cured/Cleared OR (Step 4)^2^ | |
| --- | --- | --- | --- | --- |
|  | Odds Ratio | 95% CI | Odds Ratio | 95% CI |
| FLOC | *Ref* | *Ref* | *Ref* | *Ref* |
| MI | 0.25 | **0.17 – 0.35** | 0.55 | **0.40 – 0.74** |
| AZ | 0.3 | **0.21 – 0.42** | 0.16 | **0.11 – 0.21** |
| CT | 0.5 | **0.35 – 0.70** | 0.67 | **0.51 – 0.88** |
| NV | 0.28 | **0.19 – 0.41** | 0.40 | **0.29 – 0.56** |
| PR | 0.05 | **0.03 – 0.07** | 0.19 | **0.13 – 0.28** |
| Notes:  Odds ratios compare the odds of receiving viral testing and achieving cure/clearance in each jurisdiction relative to Florida-Orange County (FLOC).   1. The N used for viral testing can be found in Supplemental Table 5, Step 2 (viral testing). The number of individuals not receiving viral testing is calculated as the difference between Step 1 (ever infected) and Step 2. 2. The N used for cure/clearance can be found in Supplemental Table 5, Step 4 (cured/cleared). The number of individuals not cured/cleared is calculated as the difference between Step 3 (initial infection) and Step 4. | | | | |
